# A second mechanism employed by artemisinins to suppress *Plasmodium falciparum* hinges on inhibition of hematin crystallization

**DOI:** 10.1074/jbc.RA120.016115

**Published:** 2020-12-02

**Authors:** Wenchuan Ma, Victoria A. Balta, Rachel West, Katy N. Newlin, Ognjen Š. Miljanić, David J. Sullivan, Peter G. Vekilov, Jeffrey D. Rimer

**Affiliations:** 1 Department of Chemical and Biomolecular Engineering, University of Houston, Houston, Texas, USA; 2 W. Harry Feinstone Department of Molecular Microbiology and Immunology, Malaria Research Institute, Johns Hopkins Bloomberg School of Public Health, Baltimore, Maryland, USA; 3 Department of Chemistry, University of Houston, Houston, Texas, USA

**Keywords:** malaria, hemozoin crystals, drug adducts, atomic force microscopy, parasites, AFM, atomic force microscopy, ARS, artesunate, ART, artemisinin, CBSO, citric buffer–saturated octanol, CQ, chloroquine, H-ART, heme–artemisinin adduct, H-ARS, heme–artesunate adduct, MS, mass spectrometry

## Abstract

Malaria is a pervasive disease that affects millions of lives each year in equatorial regions of the world. During the erythrocytic phase of the parasite life cycle, *Plasmodium falciparum* invades red blood cells, where it catabolizes hemoglobin and sequesters the released toxic heme as innocuous hemozoin crystals. Artemisinin (ART)-class drugs are activated *in vivo* by newly released heme, which creates a carbon-centered radical that markedly reduces parasite density. Radical damage to parasite lipids and proteins is perceived to be ARTs’ dominant mechanism of action. By contrast, quinoline-class antimalarials inhibit the formation of hemozoin and in this way suppress heme detoxification. Here, we combine malaria parasite assays and scanning probe microscopy of growing β-hematin crystals to elucidate an unexpected mechanism employed by two widely administered antimalarials, ART, and artesunate to subdue the erythrocytic phase of the parasite life cycle. We demonstrate that heme–drug adducts, produced after the radical activation of ARTs and largely believed to be benign bystanders, potently kills *P. falciparum* at low exogenous concentrations. We show that these adducts inhibit β-hematin crystallization and heme detoxification, a pathway which complements the deleterious effect of radicals generated *via* parent drug activation. Our findings reveal an irreversible mechanism of heme–ART adduct inhibition of heme crystallization, unique among antimalarials and common crystal growth inhibitors, that opens new avenues for evaluating drug dosing regimens and understanding growing resistance of *P. falciparum* to ART.

The increased number of malaria deaths last century attributed to quinoline resistance (1) has been reversed by the implementation of effective artemisinin (ART) combination therapies (2, 3). ART-class drugs are activated by reduced heme, a byproduct of hemoglobin endocytosis and catabolism within the malaria parasite. Resulting cleavage of the endoperoxide bridge generates a free radical that damages vital parasite proteins and lipids (4, 5), with a concomitant 10,000-fold reduction in parasite density in human patients (6). Recently, a delayed clearance phenotype (7) deemed resistance to ARTs has emerged in Southeast Asia and has been linked, *via* an altered Kelch13 gene (8), to decreased hemoglobin supply and lower drug activation at the ring stage of the parasite life cycle, leading to a marked tolerance of ring stage parasites to ARTs (9, 10). Parasites in the subsequent trophozoite stage are susceptible to activated ARTs (11, 12), yet the mechanism of retained trophozoite sensitivity has been elusive.

The signature endoperoxide group of ART-class drugs, such as ART (Fig. 1*A*) and artesunate (ARS, Fig. 1*D*), is activated by reduced heme iron(II) to generate a carbon-centered radical (Fig. 1*B*), which can alkylate and damage nearby *Plasmodium* proteins and lipids (4). This suicide activation imparts a rapid 10,000-fold drop in parasite density over the first 48 h (6). The radicals terminate by alkylating heme to generate respective heme(III)-drug adducts (heme–artemisinin adduct [H-ART] and heme–artesunate adduct [H-ARS] in Fig. 1, *C* and *E*, respectively), which have been considered to be correlative biomarkers of the activity of ART-class drugs and potential inhibitors of hemozoin (13, 14, 15). The adduct’s redox active heme iron was also proposed to continue to cause radical damage to contribute to toxicity (16, 17). Parasites treated with radiolabeled [^14^C] ART accumulate H-ART, retaining 75% of the applied drug in close association with hemozoin (18). Similarly, H-ARTs were found in the spleens and urine of ART-treated *Plasmodium* infected mice (19). Analyses of 10-deoxodihydroartemisinin which cannot easily undergo ring opening versus dihydroartemisinin indicates ring opening correlated with heme crystal inhibition (20); however, reducing conditions favoring heme(II) lead to the generation of heme–drug adduct, which has been proposed to suppress hemozoin growth (21, 22). H-ARTs were detected in *Plasmodium falciparum* cultures at ring, trophozoite, and schizont stages; notably, the Kelch13 mutants, associated with the delayed clearance phenotype, displayed lower adduct concentrations (23). None of these findings address the exact role of ART-class adducts and their mechanism of action on both ring and trophozoite stage parasites.

**Figure 1:**
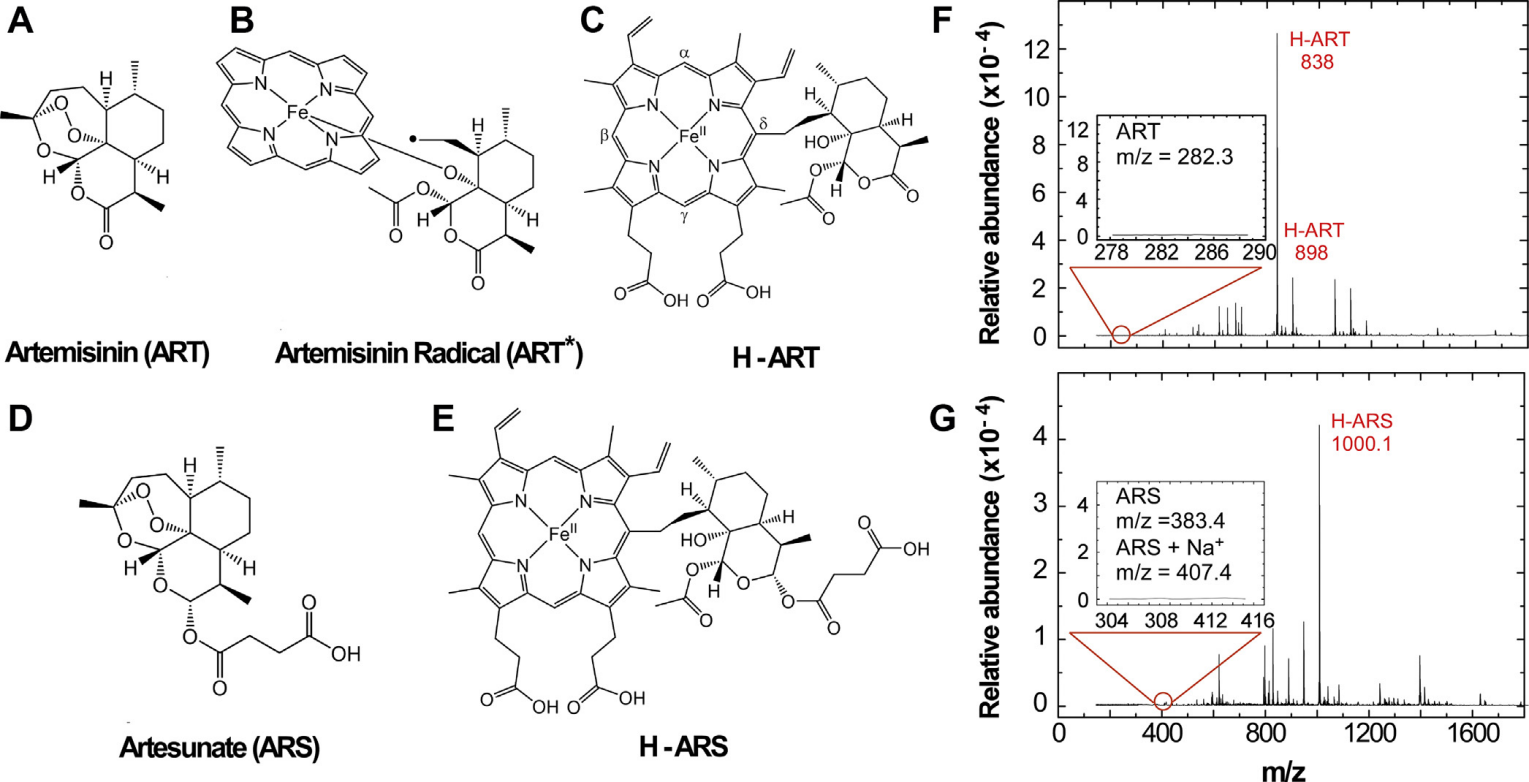
***In vitro* activation of artemisinins to heme–drug adducts.***A–E*, molecular structures of (*A*) ART, (*B*) the radical ART∗ generated from the reaction between ART and heme (II) (Fig. S1), and (*C*) the adduct H-ART formed between ART∗ and heme(III). Carbons-labeled α, β, δ, and γ indicate sites for isomorphic ART∗ substitutions. The chemical bond is arbitrarily shown at the δ position (noting that all positions can be alkylated). *D* and *E*, molecular structures of ARS, in (*D*), and its adduct (H-ARS), in (*E*), generated by a similar reaction mechanism (Fig. S2). *F*, mass spectrum of isolated H-ART with characteristic peaks at *m*/*z* = 838 and 898. *Inset*: absence of mass ion peaks associated with the parent drug ART. *G*, mass spectrum of isolated H-ARS with its characteristic peak at *m*/*z* = 1000.1. *Inset*: absence of mass ion peaks associated with the parent drug ARS. ARS, artesunate; ART, artemisinin; H-ART, heme–artemisinin adduct; H-ARS, heme–artesunate adduct.

Here, we design an *in vitro* protocol to synthesize the heme–drug adducts of ART-class drugs (Fig. S1) and test their activity in suppressing *P. falciparum* and the associated molecular mechanisms targeting heme crystallization. We place ART or ARS in contact with heme(III) and a reducing agent (dithionite) in a biphasic butanol-water solvent. The resulting reactions (Fig. S2) and purification by chromatography (Fig. S3) lead to the heme–drug adduct in ∼50% yield. Mass spectrometry (MS) identified the characteristic mass-to-charge (*m*/*z*) ratios of H-ART as 838 and 898 (Fig. 1*F*) and of H-ARS as 1000.1 (Fig. 1*G*); the *m*/*z* values for H-ART (Fig. 1*G*) are similar to those of H-ART extracted from the spleens of malaria-infected mice treated with ART (19). MS spectra also revealed the absence of residual ART (*m*/*z* = 282.3, inset of Fig. 1*F*) and ARS (*m*/*z* = 383.4, inset of Fig. 1*G*). These analyses confirm the synthesis of adducts H-ART and H-ARS, which both lack functional endoperoxides.

We added known quantities of heme–drug adducts (Fig. S4) to parasite cultures of three *P. falciparum* strains: chloroquine (CQ) and ART-sensitive NF54, CQ-resistant and ART-sensitive CamWT, and CQ and ART-resistant C580Y. Traditional 72-h continuous IC_50_ assays (24) were performed using radiolabeled hypoxanthine [^3^H] incorporation to assess the impact of adducts and their corresponding parent drugs on parasite growth (Fig. 2, *A* and *B*). The resulting IC_50_ values are below 10 nM for both H-ARS and H-ART (Fig. 2*C*), similar to the IC_50_ of ART and ARS alone. The low nanomolar IC_50_ values for H-ARS and H-ART suggest an independent mechanism of action from the radical damage caused by the parent drug. To assess ring stage survival of the three *P. falciparum* strains treated with parent drugs and corresponding heme–drug adducts, we pulsed 500 nM of H-ARS or H-ART for 6 h at ring and trophozoite stages in parallel with the same concentration of parent drug (ARS or ART) and CQ, which was used as a reference. Interestingly, the C580Y isolate had a survival rate of 21% when exposed to H-ARS compared with 42% for the parent drug (Fig. 2*D*), whereas values for H-ART, ART, and CQ were similar within experimental error. H-ART and ART result in approximately 17% survival in NF54 and CamWT, which both lack the mutant Kelch 13 gene of C580Y. A short pulse of CQ was effective against CQ-sensitive NF54, but more than 60% of parasites survived in the CQ-resistant CamWT and C580Y isolates. A 6-h pulse at the trophozoite stage killed 95% of all three isolates for all five drugs.

**Figure 2:**
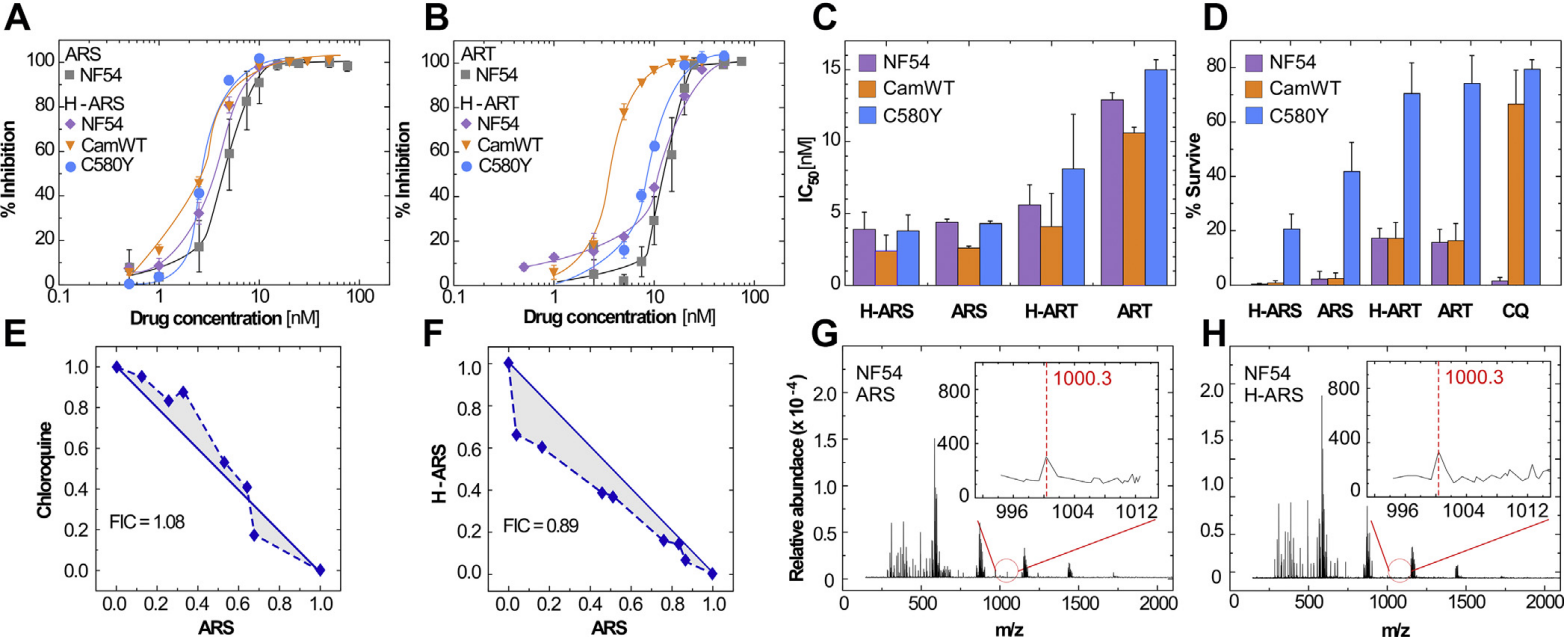
***P. falciparum* inhibition by heme–drug adducts.***A* and *B*, percent inhibition of three *P. falciparum* strains, NF54, C580Y, and CamWT, after 72 h of continuous drug (parent or adduct) exposure as a function of drug concentration: (*A*) ARS and H-ARS; (*B*) ART and H-ART. *C*, IC_50_ assays were performed with each *P. falciparum* isolate for the parent drugs (number of biological replicates, n = 4) and adducts H-ART (n = 3) and H-ARS (n = 5). *D*, results of ring stage survival assays with 500 nM of drugs pulsed for 6 h, then washed, and incubated for an additional 66 h compared with assays without drug (n = 3). *E* and *F*, isobolograms for combination drugs with an outcome of IC_50_ and corresponding fractional inhibitory concentrations (FICs): (*E*) CQ and ARS; (*F*) H-ARS and ARS. *G* and *H*, MS spectra of extracted hemozoin crystals after a 6-h incubation of trophozoites with ARS in (G) and H-ARS in (H). The samples were frozen and hemozoin was pelleted and decrystallized (for details of the extraction procedure and expanded MS analysis, see the Methods in the Supporting Information and Fig. S5). ART, artemisinin; ARS, artesunate; CQ, chloroquine; H-ART, heme–artemisinin adduct; H-ARS, heme–artesunate adduct; MS, mass spectrometry.

Antimalarials are often administered as combination therapies to achieve synergism; however, recent findings have provided examples where certain combinations of drugs can give rise to antagonistic cooperativity (25). Construction of isolobolograms helps distinguish the cooperativity of drug combinations, which were used here to test binary mixtures of ARS with CQ (Fig. 2*E*) and H-ARS (Fig. 2*F*). *P. falciparum* culture with the former results in no observable changes off the line of addition, indicating additivity. The combination of ARS and H-ARS, however, shows a change from strict additivity to mild synergistic cooperativity. This finding indicates that activation of ARS to H-ARS leads to enhanced efficacy, which is consistent with IC_50_ assays (Fig. 2*C*). We tested whether heme–drug adducts directly associate with hemozoin crystals in *P. falciparum* culture. We pulsed NF54 at the trophozoite stage for 6 h with 500 nM ARS (Fig. 2*G*) and H-ARS (Fig. 2*H*) in comparison to a control (without drug). Hemozoin was extracted from the parasite to assess residual compounds adsorbed on crystal surfaces. Analysis by MS revealed no remnants of the parent drug (ARS), whereas spectra of extracts from isolates exposed to parent drugs or heme–drug adducts contained peaks corresponding to either H-ARS (insets of Fig. 2, *G* and *H*) or H-ART (Fig. S5). These findings provide further evidence of heme–drug adduct formation *in vivo* and their association with hemozoin crystals.

Molecular confirmation of drug–crystal association and its impact on crystal growth inhibition was obtained using time-resolved *in situ* atomic force microscopy (AFM) to monitor β-hematin surface growth in a biomimetic solution containing supersaturated heme (26) in the presence or absence of antimalarial drugs. Focusing on the basal (100) face, we observe the presence of unfinished layers with heights *h* = 1.17 ± 0.07 nm (Fig. 3*A*), close to the relevant unit cell dimension (*a* = 1.22 nm) (27). The dynamics of surface growth captured by AFM reveals a classical layer-by-layer mechanism wherein islands nucleate and grow by the attachment of solute molecules to steps (26). Analysis of *in situ* AFM images permits the determination of two variables used to assess the efficacy of antimalarials: the rate of layer nucleation, *J*_*2D*_, as the number of islands that nucleate per unit area per time, and the velocity, *v*, of advancing steps (28). We recently examined the impact of quinoline-class antimalarials on β-hematin surface growth and observed distinct modes of drug–crystal binding (26). One of the most prominent mechanisms of crystal growth inhibition is step pinning, which occurs when drugs bind to terrace sites on the crystal surface and impose a curvature (*i.e.,* surface tension) on advancing steps that can lead to growth succession at appreciable drug concentration. A second mode of growth inhibition is kink blocking (29, 30), which occurs when drug molecules bind to the most favorable sites on the crystal surfaces for solute incorporation (kink sites) and markedly slow, but not fully suppress, the rate of growth.

**Figure 3:**
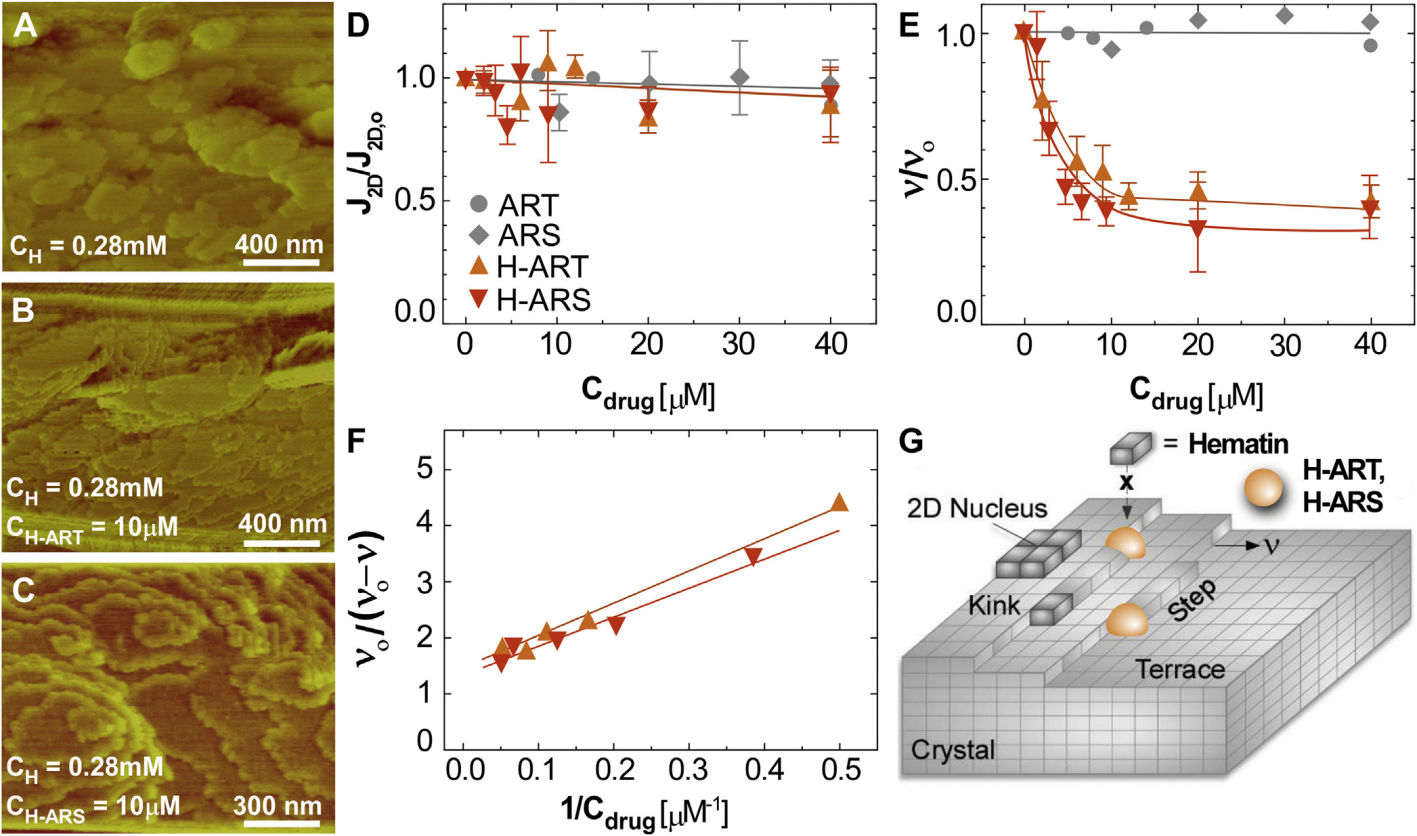
**AFM analysis of parent drug and heme–drug adduct inhibition of β-hematin growth.***A*, AFM deflection mode image of a β-hematin (100) surface in supersaturated solution with heme concentration C_H_ = 0.28 mM and at 28 °C, the temperature of the AFM liquid cell. *B* and *C*, images of an identical surface in the presence of 10 μM growth inhibitors: (*B*) H-ART and (C) H-ARS. *D*, measurements of the rate of two-dimensional nucleation, *J*_*2D*_, relative to that in the absence of any inhibitor, *J*_*2D,o*_, as a function of drug concentration. *E*, measurements of step velocity, *v*, in the [001] direction with increasing drug concentration relative to the value measured in the absence of drug, *v*_o_. *F*, linearized step velocity coordinates based on a reported equation (28) used to confirm crystal growth inhibition by a kink blocking mode of action. *G*, illustration of a crystal surface highlighting different sites for solute/drug binding (kinks, steps, and terraces) where both H-ART and H-ARS (*orange spheres*) act as kink blockers. AFM, atomic force microscopy; ART, artemisinin; ARS, artesunate; H-ART, heme–artemisinin adduct; H-ARS, heme–artesunate adduct.

The AFM images during growth assays in the presence of H-ART (Fig. 3*B*) and H-ARS (Fig. 3*C*) reveal virtually no change in the rate of layer nucleation with increasing drug concentration when scaled by the value in the absence of drug, *J*_*2D,o*_ (Fig. 3*D*). In contrast, there is a noticeable reduction in step velocity in the presence of drugs relative to the control (*i.e.,* step velocity in the absence of drugs, *v*_o_), as shown in Fig. 3*E*. Interestingly, the pristine (or inactivated) forms of artemisinins, ART and ARS, have no observable effect on β-hematin surface growth (4, 31, 32, 33), whereas their activated forms (H-ART and H-ARS) are effective growth modifiers that reduce step velocity by nearly 60%. The step velocity *versus* drug concentration profiles for both heme–drug adducts are signatures of a kink blocking mechanism, which was verified by replotting the data in the well-known Bliznakov coordinates (28) (Fig. 3*F*), where the linearized data confirm that adducts preferentially bind to kink sites on β-hematin surfaces (as illustrated in Fig. 3*G*). This finding provides direct evidence that heme–drug adducts operate as crystal growth inhibitors, in agreement with previous suggestions by Leiserowitz and Weissbuch (13).

The idealized scheme in Fig. 4*A* depicts the erythrocyte stage of the *P. falciparum* life cycle. Chronic asexual replication is depicted in optical micrographs of *P. falciparum* cultures showing the early ring stage (Fig. 4*A*, I) followed by the trophozoite stage (Fig. 4*A*, II), which is marked by parasite growth and a change in morphology. During this period there is a breakdown of hemoglobin, generating free heme (Fig. 4*B*) and initiating the formation and accumulation of hemozoin crystals. Nuclear division of trophozoites signals the onset of the schizont stage (Fig. 4*A*, III). *P. falciparum* cultures with known Kelch mutations have shown sensitivity to ART in the early ring and trophozoite stages (Fig. 4*B*, *red shaded regions*). Given that hemoglobin digestion is highest during the trophozoite stage, it is expected that H-ART would be most prevalent during this period where we have demonstrated its effective inhibition of hemozoin formation. Interestingly, *in vitro* assays reveal the heme–drug adduct operates by a unique mode of action wherein exposure of β-hematin crystals to H-ART leads to irreversible inhibition, such that removal of H-ART does not lead to the recovery of crystal growth. Direct evidence for irreversible inhibition was obtained by monitoring the dynamic response of β-hematin crystal surfaces and the velocity of step advancement in the presence of H-ART and after its removal from the solution (Fig. 4*C*). Time-resolved *in situ* AFM measurements were conducted using concentrations of H-ART and heme estimated from *P. falciparum* cultures: 10 μM H-ART is comparable to the dihydroartemisinin concentration found in trophozoites exposed to dihydroartemisinin (23), and 0.28 mM heme is comparable to the concentration of soluble heme in DCPC580Y trophozoites (34). At these conditions, H-ART results in ca. 40% reduction in step velocity (Fig. 4*C*, stage 2). Holding H-ART concentration fixed, we increased the concentration of heme to 0.50 mM to simulate an escalation in free heme during the trophozoite stage because of the combined actions of hemoglobin digestion (Fig. 4*B*) and reduced heme detoxification by H-ART crystal growth inhibition. This elevated heme concentration promotes β-hematin crystallization in the growth medium (35), resulting in the deposition of nanocrystals (ca. 200 nm) on the surface (*arrow* in Fig. 4*D*). While increased supersaturation is expected to result in higher growth rates, the nanocrystalline deposits inhibit the propagation of adjacent layers, leading to no observable change in step velocity (Fig. 4*C*, stage 3). This level of crystal growth inhibition was maintained even upon removal of H-ART from the growth solution (Fig. 4*C*, stages 4 and 5), signifying irreversible inhibition of step growth as a result of the nanocrystalline deposits.

**Figure 4:**
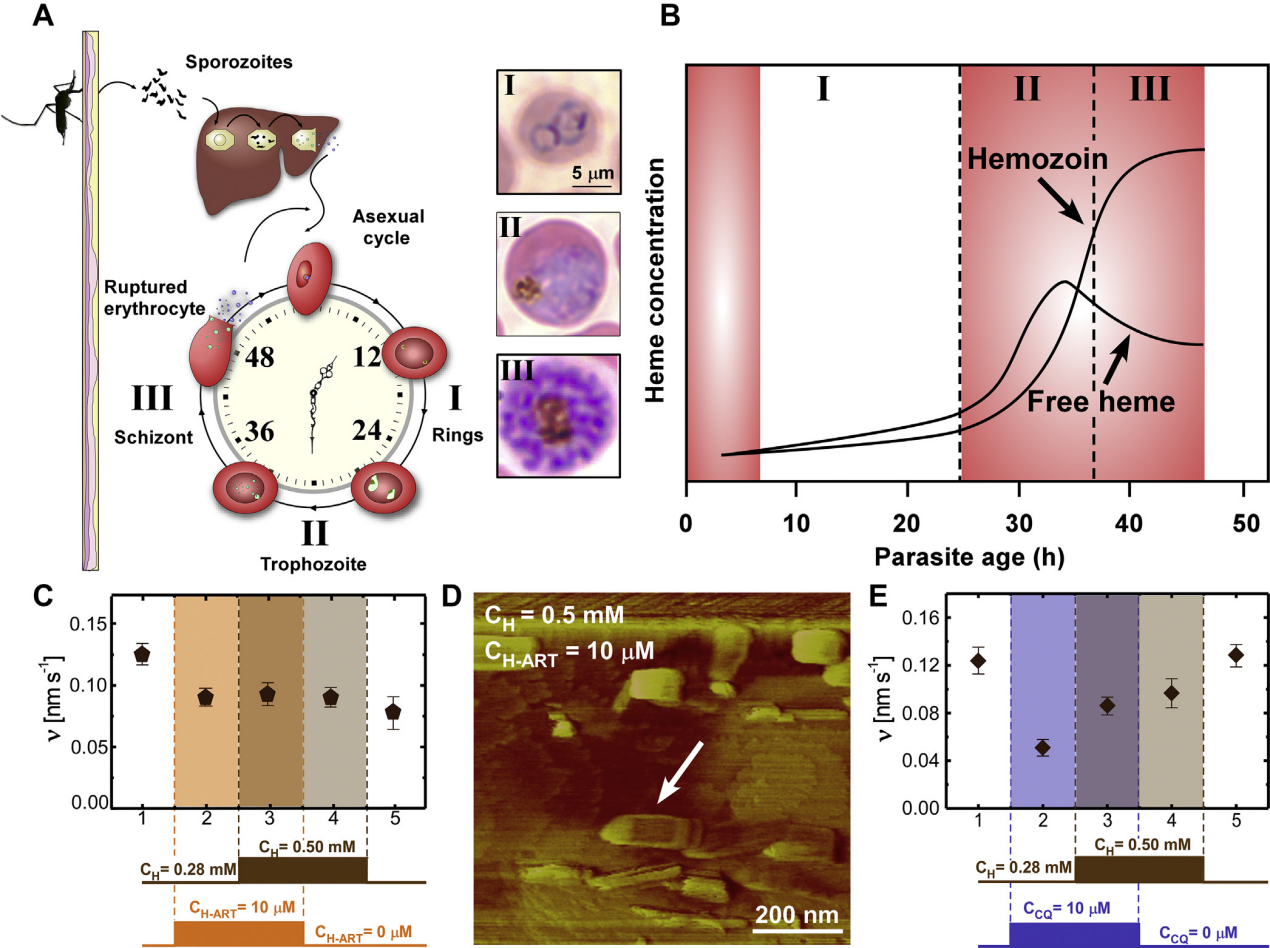
**Dual action mechanism of artemisinins.***A*, illustrated erythrocytic life cycle of *P. falciparum*. Optical micrographs represent *P. falciparum* parasites at ring (I), trophozoite (II), and schizont (III) stages. *B*, idealized scheme of hemozoin accumulation and free heme concentration variations during the parasite life cycle. Concentration profiles are adapted from data presented by Heller and Roepe (34). The *shaded red regions* correspond to the approximate times reported by Klonis *et al*. (10), where *P. falciparum* is most sensitive to 4-h pulses of ART. *C–E*, staged *in situ* AFM measurements of β-hematin surface growth at different combinations of heme and drug concentrations. Step velocities are measured in the absence of drug (stage 1); in the presence of 10 μM (*C*) H-ART or (*E*) CQ (stages 2 and 3); at increased heme concentration, 0.50 mM, in the presence (stage 3) and absence (stage 4) of drug; and again at the initial heme concentration, 0.28 mM, in the absence of drug (stage 5). *D*, AFM deflection mode image of a β-hematin (100) surface during stage 3 analysis showing the presence of deposited nanocrystals (*arrow*). AFM, atomic force microscopy; ART, artemisinin; CQ, chloroquine; H-ART, heme–artemisinin adduct.

For comparison, a similar staged *in situ* AFM experiment was performed with CQ (Fig. 4*E*). Unlike H-ART, a step increase in heme concentration in the presence of CQ did not lead to any detectable generation of β-hematin crystals in the growth medium. Moreover, increased supersaturation resulted in a higher step velocity (Fig. 4*E*, stage 3), whereas the removal of CQ from the growth solution resulted in a progressive increase in step velocity such that a full recovery to the initiate state was observed within 2 to 3 h of continuous imaging (Fig. 4*E*, stage 5). This indicates that the action of CQ is fully reversible, in stark contrast to the unique behavior of H-ART. These findings incite new questions regarding the generality of irreversible inhibition among ART-class drugs and how this additional mode of action could potentially alter ART dosing regimens.

In summary, we address the prevailing hypothesis that ART-class drugs exhibit a singular mode of action involving suicide activation and radical damage. Indeed, we show that adducts relegated as biomarkers of ARTs’ activation operate by a second heme crystal inhibition mode of action during the trophozoite stage of the parasite life cycle. The efficacy and mechanism of heme–drug adducts synthesized with two ARTs were tested by a combination of *P. falciparum* cultures and *in situ* AFM measurements of β-hematin crystallization. Our findings reveal that both adducts efficiently kill parasites in cultures. We have not addressed a potential third mechanism of action where the heme–drug adduct becomes a redox active molecule which continuously generates radicals instead of the single suicide action of the parent drug proposed by Roberts *et al.* (16, 17). For ART-resistant strains, adducts are more effective than their respective parent drugs at lower doses. Analysis of hemozoin crystals isolated from parasites treated with ART-class drugs demonstrates that adducts H-ART or H-ARS are associated with the crystals, consistent with time-resolved measurements of β-hematin crystallization with varied heme and drug concentrations, revealing a unique mode of irreversible crystal growth inhibition. This unanticipated mechanism has potential implications for understanding heme detoxification *in vivo*, such as the ability of heme–drug adducts to force free heme concentrations above the toxicity limit after depletion of the drug in resistant parasites. Retained suppression of heme detoxification after drug removal is a direct way to circumvent resistance to ARTs *via* a mechanism that is distinctly unique compared with those of quinoline-class antimalarials.

## Experimental procedures

A summary of experimental procedures is provided below with additional details included in the Supporting Information.

### Growth solution preparation

Citric buffer at pH 4.8 was prepared by dissolving 50 mM of citric acid in deionized water and titrating the solution, under continuous stirring, with the addition of 0.10 M NaOH to reach the desired pH. Fresh buffers were prepared every month and stored at ambient conditions. We placed 5 ml of citric buffer (pH 4.8) in direct contact with *n*-octanol at room temperature and allowed 30 min for equilibration. The upper portion of the two-phase system was decanted and denoted as citric buffer–saturated octanol (CBSO). Heme solutions were prepared by dissolving heme powder in 8 ml of freshly made CBSO and heating to 70 °C for 7 to 9 h. The solution was filtered, and the concentration was determined by UV–Vis spectroscopy.

### Synthesis of heme–drug adducts

Heme solutions were prepared by a modified method using the same procedure as CBSO, but *via* substitution of *n*-octanol with *n*-butanol (Fig. S1). Sodium dithionite and ART were dissolved in DI water and *n*-butanol, respectively. The heme solution was filtered and placed in contact with the dithionite solution in a glass vial to yield a net molar ratio of 1:2:5 heme: ART: sodium dithionite. The vial was sealed under flow of nitrogen gas to create an inert atmosphere. The reaction involved the reduction of heme(III) to heme(II) with dithionite acting as the reducing agent. The system was maintained at 50 °C using a water bath. The aqueous and organic phases were rigorously mixed by shaking for ca. 30 s until the color of the solution changed from dark green to pink, indicating the reduction of heme(III) to heme(II). The mixture was kept under static conditions for at least 30 min to allow for the separation of organic and aqueous phases, after which the ART solution was injected into the organic (top) fraction. The reaction between heme(II) and ART happened immediately after the injection, as surmised by the instantaneous change in color from pink to orange. After allowing ca. 30 min for the reaction to reach completion, the organic layer was collected for later purification of the product, H-ART. The procedure for synthesizing H-ARS was identical to that of H-ART with the replacement of ART with ARS. A detailed reaction mechanism of heme–drug adduct generation is provided in Fig. S2.

### Purification of heme–drug adducts

The collected organic fraction from the biphasic reaction was first passed through a 0.2 μm filter before injecting the solution into a HPLC system equipped with a C18 column and two UV-Vis detectors with absorption wavelengths set at 215 nm to detect unreacted parent drug (ART and ARS) and 470 nm to detect the heme–drug adducts (H-ART and H-ARS) as well as unreacted heme(III). For the separation of heme–drug adduct from the reaction mixture, we used a mobile phase with a composition of 5% methanol, 45% acetonitrile, and a 50% mixture of formic acid (0.1%) in DI water (note that percentages are based on mass). Unreacted heme(III) elutes at a retention time of *t* = 23.5 min (Fig. S3), whereas both adducts (H-ART and H-ARS) elute at a nearly equivalent retention time of *t* ≈ 5.8 min.

The concentration of the collected heme–drug adducts was quantified using UV-Vis adsorption https://uh.edu/tech/clist/_documents/manuals/computer-controlled-equipment/du800one.pdf. The extinction coefficient of heme dissolved in a dimethyl sulfoxide solution at λ = 400 nm was determined with standard solutions at fixed concentration. A representative spectrum of each compound and the calibration curve used to measure the extinction coefficient are provided in Fig. S4, *A* and *B*, respectively.

### Preparation of heme crystal substrates

A heme growth solution was prepared by dissolving hematin powder in 8 ml of freshly made CBSO followed by a 7 to 9 h period of heating at 70 °C. The solution was cooled to room temperature under ambient conditions and filtered with a 0.2 μm nylon membrane filter. The concentration was determined with an extinction coefficient $\varepsilon_{heme}$ (Fig. S4*B*). The hematin solution was then diluted with fresh CBSO to achieve a final concentration of 0.20 μM. A piece of cover glass, cleaned with multiple water–ethanol–water cycles, was placed at the bottom of a glass vial and immersed in heme growth solution. Glass vials were sealed with closed-top septa caps and stored on a stationary platform in the dark at room temperature. Small crystals appeared on the glass slides after 2 to 3 days and reached a maximum size (ca. 20 μm) after 2 weeks.

### *In situ* monitoring of heme crystal growth

The AFM images were collected in tapping mode (*i.e.,* light engage) with a frequency of 32 kHz. The density of heme crystal substrates grown on glass disks (as described above) was monitored with an optical microscope to ensure an equivalent number of crystals for all samples (*i.e*., minimize potential depletion of free heme and growth inhibitor because of high total surface area of crystals). The glass slides were mounted on AFM sample disks, and the samples were placed on the AFM scanner. Supersaturated heme solutions in CBSO were prepared less than 2 h in advance. The growth solution was loaded into the AFM liquid cell using a disposable polypropylene syringe. After loading, the system was left standing for 10 to 20 min to thermally equilibrate.

The solution in the AFM fluid cell was exchanged every 30 min to maintain an approximately constant heme (and inhibitor) concentration. For studies of antimalarials, growth solutions were replaced with ones containing a selected drug concentration. For each assay, the crystal substrates were first allowed to equilibrate (ca. 30 min) in growth solution without added drug before addition of solutions containing the drug. For studies assessing irreversible inhibition, a series of solutions with varying heme and/or drug concentrations were supplied to the AFM liquid cell at periodic imaging times. For all *in situ* measurements, the growth of heme crystal surfaces *via* two-dimensional layer generation and spreading was characterized by the velocity of step advancement *v* (nm/s) and the rate of two-dimensional nucleation of new crystal layers *J*_*2D*_ (nm^-2^s^-1^) using reported protocols (28). In brief, we determine *v* by monitoring the displacements of 8 to 13 individual steps with a measured step height h = 1.17 ± 0.07 nm (corresponding to the unit cell dimension in the *a*-direction). Approximately 25 to 35 measurements were taken for each individual step, and the average growth rates were reported by analysis of sequential images over time. To determine *J*_*2D*_, the appearance of new islands on the surface between successive images was monitored, and the number of islands that grew was counted. This number was scaled with the scan area and the time interval between images to yield *J*_*2D*_ (assessed from the average of 15–25 measurements).

### *In vitro P. falciparum* inhibitory drug assay

*P. falciparum* cultures were synchronized to the ring stage by incubation in 5% sorbitol. Parasitemia was assessed by optical microscopy of a Giemsa-stained blood film. The IC_50_ value was determined using a modified version of the [^3^H]-hypoxanthine incorporation assay. Each drug concentration was performed in three technical replicates. Negative growth control wells contained 10 μM of CQ. Positive growth control wells contained drug-free culture media. Parasite cultures were plated in tissue culture treated 96-well plates at 2% hematocrit and 0.5% parasitemia in a final volume of 200 μl of hypoxanthine-free complete media. Parasite cultures were incubated with drug continuously for 72 h. At the time of incubation with drug, 0.5 μCi of [^3^H]-hypoxanthine was added to each well. Upon completion of the incubation period, 96-well plates were frozen at –80 °C until ready for sample harvesting. The 96-well plates were thawed, and samples were harvested onto glass fiber filters by a cell harvester. Incorporation of [^3^H]-hypoxanthine was measured on a liquid scintillation counter. Parasite growth was determined by comparing the disintegrations per minute of control wells to test wells. IC_50_ curves were generated by nonlinear regression analysis. Isobologram analysis was performed with a checkerboard at three or more concentrations above the IC_50_ and three or more below the IC_50_ value. The IC_50_ was determined for each drug alone and in mixed concentration ratios. The individual and sum 50% fractional inhibitory concentrations were determined. Isobolograms were plotted from the FIC_50_s of drug 1 and drug 2 at the tested fractions of the IC_50_.

### Ring stage assay

*P. falciparum* cultures were synchronized to the ring stage by incubation in 5% sorbitol 48 h before and immediately before drug pulse. Drugs were added at 500 nM for 6 h and washed thrice in culture media without hypoxanthine and then returned to complete media for another 66 h with 0.5 μCi of [^3^H]-hypoxanthine added to each well.

### Hemozoin isolation

*P. falciparum* cultures were synchronized to the ring stage by incubation in 5% sorbitol. After 24 h progression to the trophozoite stage, drugs were added at 500 nM for 6 h. The parasites were harvested by saponin lysis and frozen at a temperature of –80 °C. Parasites were resuspended in 0.2% SDS/100 mM bicarbonate at pH 10 and centrifuged. Parasites were then resuspended in 100 mM bicarbonate pH 10 and centrifuged. The hemozoin pellets were then washed at 4 times in DI water. Frozen hemozoin was then *decrystallized* (or dissolved) with ammonium hydroxide before analysis.

## Data availability

All data are included in this article. Raw datasets are available upon request.

## Conflict of interest

The authors declare that they have no conflicts of interest with the contents of this article.
