# Supplementary material for: A second mechanism employed by artemisinins to suppress Plasmodium falciparum hinges on inhibition of hematin crystallization
Source: J Biol Chem. 2020 Dec 2;296:100123. doi: 10.1074/jbc.RA120.016115 (PMC7949059; doi:10.1074/jbc.RA120.016115)
Supplement: Figs. S1–S5 [file mmc1.pdf]

Supporting Information for

Artemisinins Deploy a Dual Mode of Therapeutic Action against *Plasmodium Falciparum*

Wenchuan Ma[1,†], Victoria A. Balta[2,†], Rachel West[2], Katy N. Newlin[1], Ognjen Š. Miljanić[3], David J. Sullivan[2], Peter G. Vekilov[1,3], Jeffrey D. Rimer[1,3]

[1] Department of Chemical and Biomolecular Engineering, University of Houston, Houston, TX 77204 USA
[2] W. Harry Feinstone Department of Molecular Microbiology and Immunology, Malaria Research Institute, Johns Hopkins Bloomberg School of Public Health, Baltimore, MD 21205 USA
[3] Department of Chemistry, University of Houston, Houston, TX 77204 USA
† Authors contributed equally

Corresponding Authors: Jeffrey D. Rimer, Peter G. Vekilov, David J. Sullivan
Email: jrimer@central.uh.edu, vekilov@uh.edu, dsulliv7@jhmi.edu

Table of Contents

Methods………………………………………………………………………………………. S2

Supporting Figures…………………………………………………………………………..... S6

References…………………………………………………………………………….............. S11

List of Figures

Figure S1: Steps involved in the synthesis of heme – drug adducts.

Figure S2: Reaction pathway for the synthesis of heme – drug adducts.

Figure S3: Chromatograms from high-performance liquid chromatography separations

Figure S4: Spectroscopic analysis of heme and heme-drug adducts.

Figure S5: Mass spectrometry data of hemozoin crystals extracted from *P. falciparum* culture.

Methods

Materials. The following compounds were purchased from Sigma Aldrich: hematin porcine (≥98%), citric acid (anhydrous, ≥99.5%), sodium hydroxide (anhydrous, ≥98%), *n*-octanol (anhydrous, ≥99%), artemisinin (≥98%), artesunate (anhydrous, ≥98.0%), sorbitol, hypoxanthine, saponin, sodium dodecylsulfate (SDS), bicarbonate, sodium bicarbonate ($NaHCO_3$, hypoxanthine, and HEPES. The following chemical were purchased from ThermoFisher Scientific: RPMI 1640 media supplemented with L-glutamine and gentamycin. Human serum was obtained from interstate blood bank. All reagents were used as received without further purification unless otherwise noted. Deionized (DI) water was produced by a Millipore reverse osmosis ion exchange system (Rios-8 Proguard 2–MilliQ Q-guard).

Growth Solution Preparation. Citric buffer at pH 4.8 was prepared by dissolving 50 mM of citric acid in DI water and titrating the solution, under continuous stirring, with the addition of 0.10 M NaOH to reach the desired pH as verified before each experiment using an Accumet Basic pH meter (Thermofisher Scientific). Fresh buffers were prepared every month and stored at ambient conditions. We placed 5 mL of citric buffer (pH 4.8) in direct contact with *n*-octanol at room temperature and allowed 30 minutes for equilibration. The upper portion of the two-phase system was decanted and denoted as citric buffer-saturated octanol (CBSO). Heme solutions were prepared by dissolving heme powder in 8 mL of freshly made CBSO and heating to 70 °C for 7–9 hours. The solution was filtered through a 0.2 μm nylon membrane filter (ThermoFisher Scientific) and the concentration was determined using a previously reported (1) extinction coefficient of 3.1 ± 0.1 cm^{-1} mM^{-1} measured at a wavelength of λ = 594 nm.

Synthesis of Heme – Drug Adducts. Heme solutions were prepared by a modified method using the same procedure as CBSO, but via substitution of *n*-octanol with *n*-butanol. Sodium dithionite and artemisinin (ART) were dissolved in DI water and *n*-butanol, respectively. The heme solution was filtered with a 0.2 μm nylon membrane filter and placed in contact with the dithionite solution in a glass vial to yield a net molar ratio of 1:2:5 heme: ART: sodium dithionite. The vial was sealed under flow of nitrogen gas to create an inert atmosphere. The reaction involved the reduction of heme(III) to heme(II) with dithionite acting as the reducing agent (step *i* in Fig. S1). The system was maintained at 50 °C using a water bath (Super-Nuova Multi-Position Digital Stirring Hotplates). The aqueous and organic phases were rigorously mixed by shaking for ca. 30 seconds until the color of the solution changed from dark green to pink, indicating the reduction of heme(III) to heme(II). The mixture was allowed to sit under static conditions for at least 30 minutes to allow for the separation of organic and aqueous phases, after which the artemisinin solution was injected into the organic (top) fraction. The reaction between heme(II) and artemisinin (step *iii* in Fig. S1) happened immediately after the injection, as surmised by the instantaneous change in color from pink to orange. After allowing ca. 30 minutes for the reaction to reach completion, the organic layer was collected for later purification of the product, heme – artemisinin adduct (or H-ART).

The procedure for synthesizing heme – artesunate adduct (H-ARS) was identical to that of H-ART with the replacement of ART with artesunate (ARS). The same reaction procedure was used with the only noticeable difference being a faster reaction to generate H-ARS, as gleaned by a more rapid color change following the addition of ARS to the heme(II) solution. A detailed reaction mechanism of heme – drug adduct generation is provided in Fig. S2.

Purification of Heme – Drug Adducts. The collected organic fraction from the biphasic reaction was first passed through a 0.2 μm filter before injecting the solution into a high pressure liquid chromatography (HPLC) system (LC-20AD prominence liquid chromatograph, Shimadzu

Corporation) equipped with a C18 column (Luna C18(2) 5μm 250 x 4.6 mm^2, Phenomenex) and two UV-Vis detectors with absorption wavelengths set at 215 nm to detect unreacted parent drug (ART and ARS) and 470 nm to detect the heme – drug adducts (H-ART and H-ARS) as well as unreacted heme(III). For the separation of heme-drug adduct from the reaction mixture, we modified a common mobile phase reported in literature (2) using a composition of 5% methanol, 45 % acetonitrile, and a 50% mixture of formic acid (0.1%) in DI water (note that percentages are based on mass). Unreacted heme(III) elutes at a retention time of t = 23.5 minutes (Fig. S3), whereas both adducts (H-ART and H-ARS) elute at a nearly equivalent retention time of $t \approx 5.8$ minutes.

The concentration of the collected heme – drug adducts was quantified using UV-Vis adsorption with a DU 800 UV/Visible Spectrophotometer. The extinction coefficient of heme dissolved in a DMSO solution at λ = 400 nm was determined with standard solutions at fixed concentration. We used the same extinction coefficient to estimate the concentrations of both H-ART and H-ARS at wavelengths 410 and 420 nm, respectively. A representative spectrum of each compound and the calibration curve used to measure the extinction coefficient are provided in Fig. S4A and S4B, respectively.

Mass Spectrometry. The mass-to-charge (*m/z*) ratio of heme and heme – drug adducts were determined using a Bruker MicroToF ESI LC-MS at Rice University. Electrospray ionization was used with the offset voltage of 4000 V and a mobile phase of 50/50 water/acetonitrile to transfer heme (dissolved in *n*-butanol), H-ART (dissolved in *n*-butanol or methanol/acetonitrile/water), and H-ARS (dissolved in *n*-butanol or methanol/acetonitrile/water) into the electron spray ionization (ESI) source.

Preparation of Heme Crystal Substrates. A heme growth solution was prepared by dissolving hematin powder in 8 mL of freshly made CBSO followed by a 7 – 9 hour period of heating at 70 °C. The solution was cooled to room temperature under ambient conditions and filtered with a 0.2 μm nylon membrane filter. The concentration was determined with an extinction coefficient $\varepsilon_{heme} = 3.1 \pm 0.1 \text{ cm}^{-1}\text{mM}^{-1} at\ \lambda = 594 \text{ nm}$ (Fig. S4B). The hematin solution was then diluted with fresh CBSO to achieve a final concentration of 0.20 μM. A piece of cover glass, cleaned with multiple water-ethanol-water cycles, was placed at the bottom of a glass vial and immersed in heme growth solution. Glass vials were sealed with closed-top septa caps and stored on a stationary platform in the dark at room temperature. Small crystals appeared on the glass slides after 2 – 3 days and reached a maximum size (ca. 20 μm) after two weeks. The glass slides containing crystals were rinsed with ethanol and DI water and dried in air prior to analysis.

***In Situ* Monitoring of Heme Crystal Growth.** All experiments were performed with a Multimode Nanoscope IV atomic force microscope (AFM) from Digital Instruments. AFM images were collected in tapping mode (i.e. light engage) using Olympus TR800PSA probes (silicon nitride, Cr/Au coated 5/30, 0.15 N m^{-1} spring constant) with a frequency of 32 kHz. Images were obtained using scan sizes of 0.3 to 20 μm, scan rates of 0.5 to 2.5 s^{-1}, 256 scan lines, and various scan angles depending on the orientation of the crystal substrate (3). The temperature in the liquid cell reached a steady value of 27.8 ± 0.1 °C within 15 minutes of imaging (3). This value was higher than room temperature owing to heating by operation of the AFM scanner. The density of heme crystal substrates grown on glass disks (as described above) was monitored with an optical microscope to ensure an equivalent number of crystals for all samples (i.e. minimize potential depletion of free heme and growth inhibitor due to high total surface area of crystals). The glass slides were mounted on AFM sample disks (Ted Pella) and the samples were placed on the AFM scanner. Supersaturated heme solutions in CBSO were prepared less than 2 h in advance. The

growth solution was loaded into the AFM liquid cell using a 1 mL disposable polypropylene syringe (Henck Sass Wolf), which is tolerant of organic solvents. After loading, the system was left standing for 10 – 20 minutes to thermally equilibrate. The crystal edges in optical micrographs were identified to determine the orientation and the crystallographic directions on the upward-facing (100) crystal surface. The crystals were kept in contact with the solution for 0.5 – 1.5 hours to allow their surface features to adapt to the growth conditions.

The scan direction was set parallel to the [001] crystallographic direction and AFM images were collected for 3 – 5 hours. The solution in the AFM fluid cell was exchanged every 30 minutes to maintain an approximately constant heme (and inhibitor) concentration. For studies of antimalarials, growth solutions were replaced with ones containing a selected drug concentration. For each assay, the crystal substrates were first allowed to equilibrate (ca. 30 minutes) in growth solution without added drug prior to addition of solutions containing the drug. For studies assessing irreversible inhibition, a series of solutions with varying heme and/or drug concentrations were supplied to the AFM liquid cell at periodic imaging times. For all *in situ* measurements, the growth of heme crystal surfaces via 2-dimensional (2D) layer generation and spreading was characterized by the velocity of step advancement v (nm/s) and the rate of 2D nucleation of new crystal layers J_{2D} ($nm^{-2}s^{-1}$) using reported protocols (3). In brief, we determine v by monitoring the displacements of 8–13 individual steps with a measured step height h = 1.17 ± 0.07 nm (corresponding to the unit cell dimension in the *a*-direction). Approximately 25 – 35 measurements were taken for each individual step and the average growth rates were reported by analysis of sequential images over time. To determine J_{2D}, the appearance of new islands on the surface between successive images was monitored and the number of islands that grew was counted. This number was scaled with the scan area and the time interval between images to yield J_{2D} (assessed from the average of 15 – 25 measurements).

Maintenance of *P. falciparum* Culture. *In vitro* experimentation involved either *P. falciparum* NF54 (MRA-1000), *P. falciparum* CamWT (MRA-1250) or *P. falciparum* CamWT_C580Y (MRA-1251), which were obtained through BEI Resources, National Institute of Allergy and Infectious Diseases (NIAID) at the National Institutes of Health (NIH), and were previously contributed by David A. Fidock. Parasite cultures were maintained under a modification of the Trager and Jensen method (4). Specifically, parasites were cultured at 2% hematocrit in RPMI 1640 media supplemented with L-glutamine, 25 mM HEPES, 0.25% $NaHCO_3$, 0.37 mM hypoxanthine, 50 µL of 50 mg/mL gentamycin, and 10% human serum. Cultures were incubated at 37 °C in 5%CO_2/5%O_2/balance N_2.

***In Vitro P. falciparum* Inhibitory Drug Assay.** *P. falciparum* cultures were synchronized to the ring stage by incubation in 5% sorbitol. Parasitemia was assessed by optical microscopy of a Giemsa-stained blood film. The half maximal inhibitory concentration (IC_{50} value) was determined using a modified version of the [^{3}H]-hypoxanthine incorporation assay (5). Each drug concentration was performed in three technical replicates. Negative growth control wells contained 10 µM of chloroquine. Positive growth control wells contained drug-free culture media. Parasite cultures were plated in tissue culture treated 96-well plates (353072, Falcon) at 2% hematocrit and 0.5% parasitemia in a final volume of 200 µL of hypoxanthine-free complete media. Parasite cultures were incubated with drug continuously for 72 hours. At the time of incubation with drug, 0.5 µCi of [^{3}H]-hypoxanthine was added to each well. Upon completion of the incubation period, 96-well plates were frozen at –80 °C until ready for sample harvesting. The 96-well plates were thawed and samples were harvested onto glass fiber filters (GF/C, Brandel) by a cell harvester (MB48, Brandel). Incorporation of [^{3}H]-hypoxanthine was measured on a liquid scintillation counter. Parasite growth was determined by comparing the disintegrations per minute

of control wells to test wells. IC_{50} curves were generated by nonlinear regression analysis, log (inhibitor) vs. response, using the GraphPad Prism 5 software. Isobologram analysis was performed with a checkerboard at three or more concentrations above the IC_{50} and three or more below the IC_{50} value (6, 7). The IC_{50} was determined for each drug alone and in mixed concentration ratios. The individual and sum 50% fractional inhibitory concentrations were determined. Isobolograms were plotted from the FIC_{50}s of drug 1 and drug 2 at the tested fractions of the IC_{50}.

Ring Stage Assay. *P. falciparum* cultures were synchronized to the ring stage by incubation in 5% sorbitol 48 hours prior to and immediately before drug pulse. Drugs were added at 500 nM for 6 hours and washed thrice in culture media without hypoxanthine then returned to complete media for another 66 hours with 0.5 µCi of [^{3}H]-hypoxanthine added to each well.

Hemozoin Isolation and Mass Spectrometry*.* *P. falciparum* cultures were synchronized to the ring stage by incubation in 5% sorbitol. After 24 hour progression to the trophozoite stage, drugs were added at 500 nM for 6 hours. The parasites were harvested by saponin lysis and frozen at a temperature of –80 °C. Parasites were resuspended in 0.2% SDS/100 mM bicarbonate at pH 10 and centrifuged. Parasites were then resuspended in 100 mM bicarbonate pH 10 and centrifuged. The hemozoin pellets were then washed at 4 times in DI water. Frozen hemozoin was then *decrystallized* (or dissolved) with ammonium hydroxide before mass spectrometry analysis.

Supporting Figures

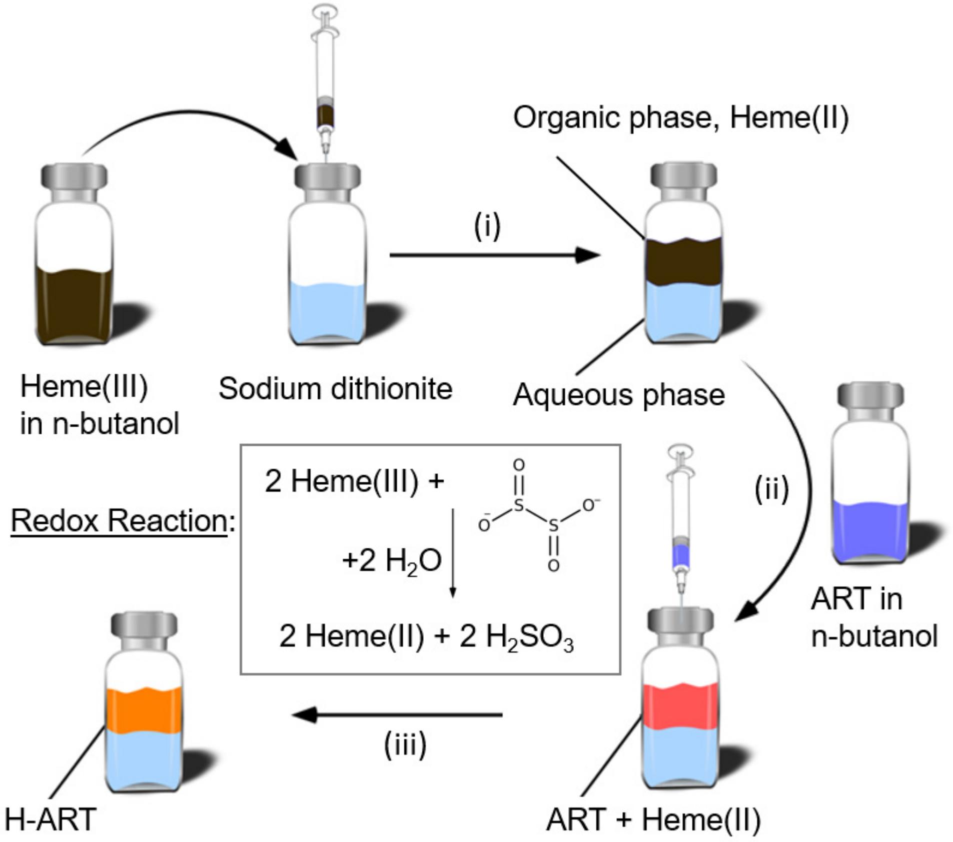

Figure S1. Steps involved in the synthesis of heme-drug adducts where we illustrate the protocol for preparing H-ART, noting that H-ARS was prepared by an identical procedure. (i) The physical mixing of heme(III) in *n*-butanol with an aqueous solution of sodium dithionite results in a biphasic solution where the reducing agent (dithionite) converts heme(III) to heme(II) by the redox reaction shown in the boxed region. (ii) To this solution is injected a mixture of drug dissolved in *n*-butanol followed by shaking. (iii) Within 30 seconds a color change in the organic phase signals ART is activated, generating H-ART via the reaction depicted in Fig. S2.

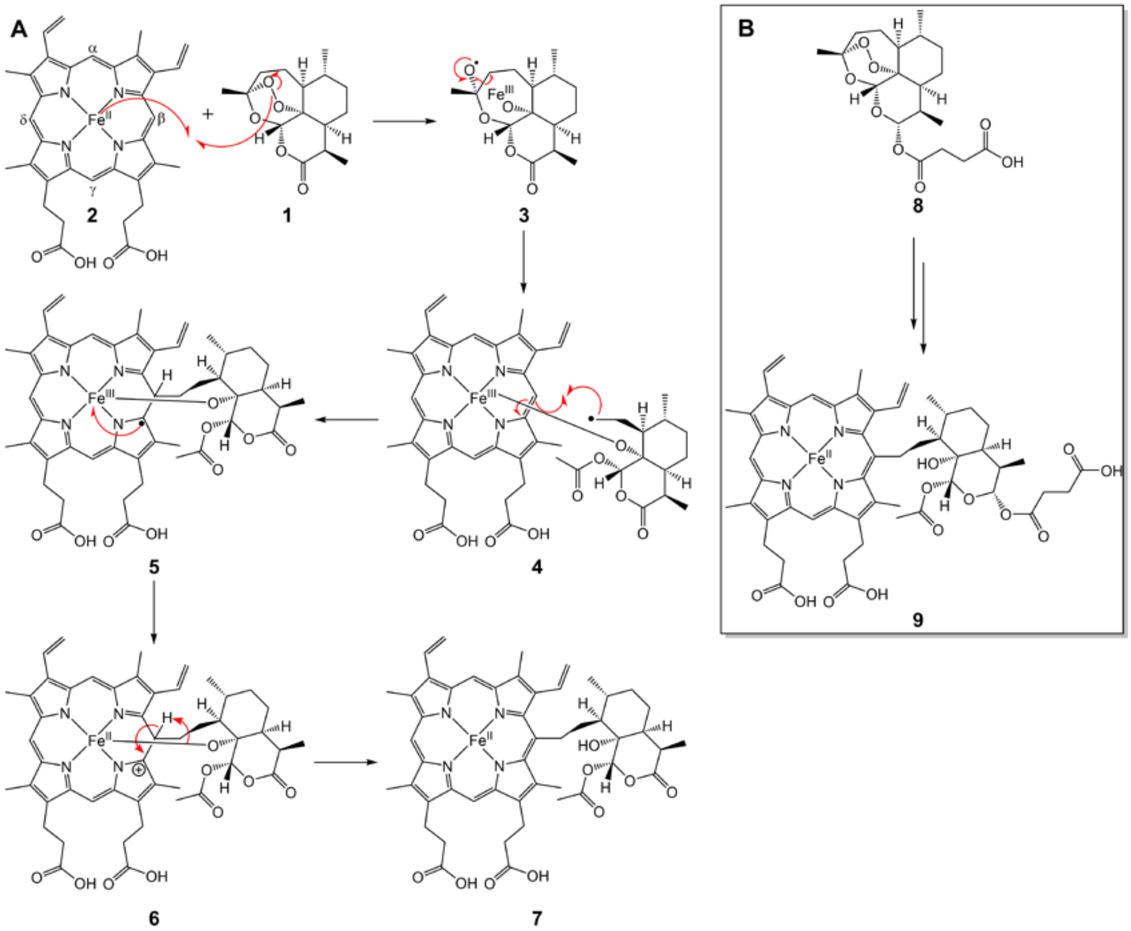

Figure S2. Reaction pathway for the synthesis of heme – drug adducts. (**A**) Mechanism of heme(II) activation of artemisinin (ART) to ART*, which rearranges and forms a covalent bond with one of the carbons of heme(III), resulting in the generation of the H-ART adduct, **7**. (**B**) Heme(II) activation of artesunate (ARS) follows the same general mechanism to yield the H-ARS adduct, **9**. In this reaction, the endoperoxide undergoes homolytic cleavage induced by heme(II), **1** – **3**. One of the oxygen atoms carries a radical center, while the other forms a covalent bond with the Fe(III) atom of heme. Once the oxygen reactive free radical **3** is generated, it propagates through the drug molecule (illustrated by red arrows), opening the seven-membered ring to create acetic ester **4**. Carbon–carbon bond formation ensues, forming **5** and placing the radical center into the five-membered ring of heme. Final electron transfer and deprotonation reestablish the conjugation in the porphyrin macrocycle.

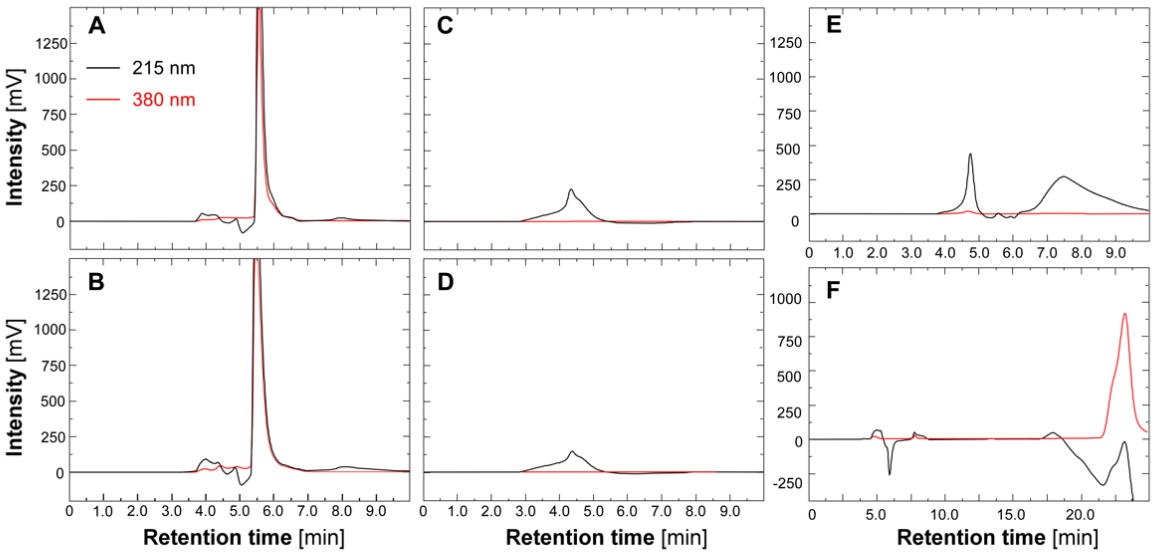

Fig. S3. Chromatograms from high-performance liquid chromatography separations showing a major peak at a retention time of 5.8 minutes for (**A**) H – ART, (**B**) H – ARS, (**C**) ART, (**D**) ARS, (**E**) sodium dithionite, and (**F**) heme. Residues of parent drugs elute at a retention time of 4.5 minutes, well separated from heme-drug adducts. The residue of heme elutes at a much later retention time of 23.5 minutes.

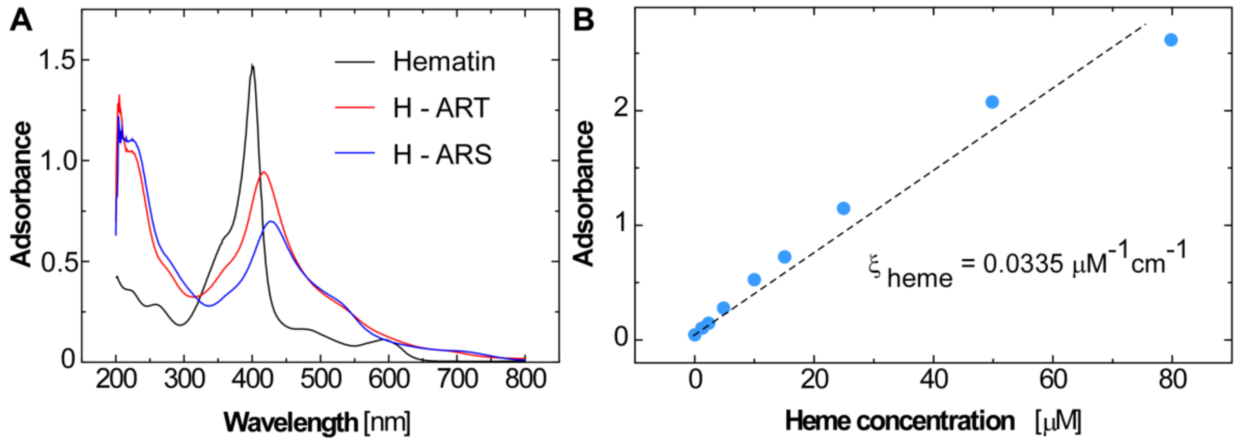

Fig. S4. Spectroscopic analysis of heme and heme-drug adducts. **(a)** UV-Vis absorption spectra of heme(III) (black line), H–ART (red line), and H–ARS (blue line) in DMSO at concentrations of 0.40, 0.23, and 0.19 mM, respectively. **b.** Calibration curve to calculate the extinction coefficient, ξ, from UV-Vis adsorption data. The absorbance at λ = 400 nm is measured at different concentrations of heme dissolved in DMSO. After the reaction, the formation of covalent bond between heme and artemisinins redistributed the π-π interactions on the porphyrin ring and the disappearance of absorbance at a wavelength λ = 594 nm indicates the loss of the methine bridge. The maximum absorbance wavelength of H–ART and H–ARS were shifted from 400 nm to 410 and 420 nm, respectively. The concentrations of purified H–ART and H–ARS were estimated according to the extinction coefficient calculated from a heme solution.

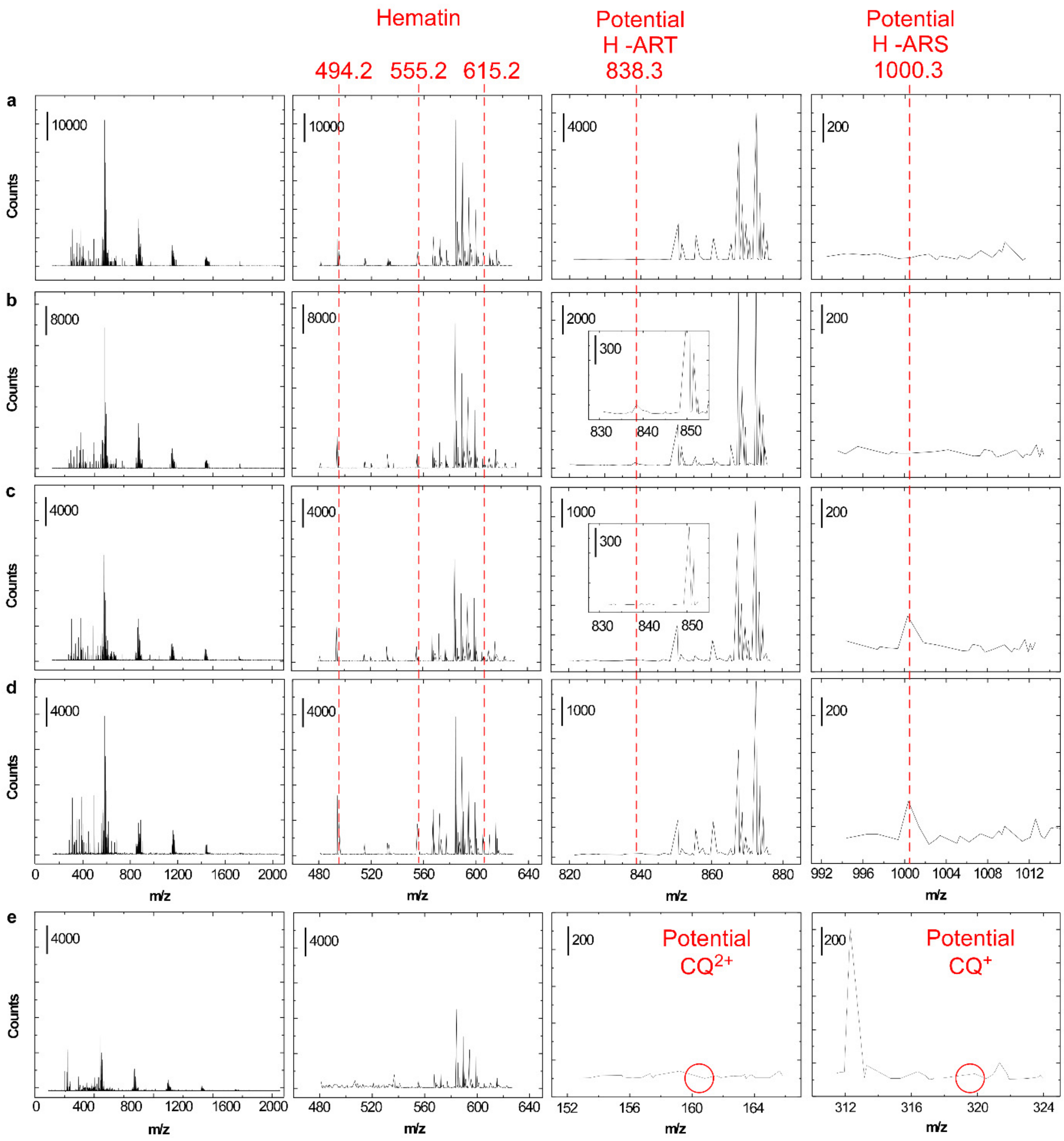

Fig. S5. Evidence of drug association with hemozoin crystals. Here we compare the mass spectrometry data of hemozoin crystals extracted from *P. falciparum* culture (NF54) after 6-hour drug pulse. Each pulse experiment used 500 nM of the following drugs: (**A**) control (no drug); (**B**) H – ART; (**C**) ARS; (**D**) H –ARS, and (**E**) choloroquine (CQ). Heme fragments at *m/z* = 615.2, 555.2, and 492.2 were detected in all samples. Scale bars for the counts are provided in each panel. Peaks at *m/z* = 838.3 for H – ART were detected in (B) and *m/z* = 1000.4 for H – ARS were detected in (C) and (D). We did not detect residual CQ in (E) at *m/z* = 319.5 for single charged CQ and at *m/z* = 159.5 for double charged CQ.

References

1. Olafson KN, Ketchum MA, Rimer JD, & Vekilov PG (2015) Mechanisms of hematin crystallization and inhibition by the antimalarial drug chloroquine. *Proc. Natl. Acad. Sci. USA* 112(16):4946-4951.
2. Stalcup AM, Martire DE, & Wise SA (1988) Thermodynamic comparison of monomeric and polymeric C18 bonded phases using aqueous methanol and acetonitrile mobile phases. *Journal of Chromatography A* 442:1-14.
3. Olafson KN, Nguyen TQ, Rimer JD, & Vekilov PG (2017) Antimalarials inhibit hematin crystallization by unique drug–surface site interactions. *Proceedings of the National Academy of Sciences* 114(29):7531.
4. Langreth SG, Jensen JB, Reese RT, & Trager W (1978) Fine Structure of Human Malaria in Vitro. *J. Protozool.* 25:443-443.
5. Desjardins RE, Canfield CJ, Haynes JD, & Chulay JD (1979) Quantitative Assessment of Anti-Malarial Activity In Vitro by a Semiautomated Microdilution Technique. *Antimicrobial Agents and Chemotherapy* 16(6):710-718.
6. Berenbaum MC (1978) Method for Testing for Synergy with Any Number of Agents. *J. Infect. Dis.* 137(2):122-130.
7. Gorka AP, Jacobs LM, & Roepe PD (2013) Cytostatic versus cytocidal profiling of quinoline drug combinations via modified fixed-ratio isobologram analysis. *Malar. J.* 12.
